# «Hanche flottante» par quelle lésion commencer?

**DOI:** 10.11604/pamj.2015.22.184.7891

**Published:** 2015-10-23

**Authors:** Soufiane Guelzim, Mohamed Saleh Berrada

**Affiliations:** 1 Service de Chirurgie Orthopédique et Traumatologie, CHU Ibn Sina, Rabat, Maroc

**Keywords:** Fractures du bassin, fractures du fémur, homolatérales, Pelvic fractures, fracture of the femur, homolateral

## Image en medicine

Les fractures ipsilatérales pelviennes et fémorales sont appelées «Hanche flottante». Il y a deux entités lésionnelles selon le type de la fracture pelvienne: fracture de l'anneau pelvien ou fracture du cotyle. Ces traumatismes de haute énergie très peu décrits en littérature posent un problème de hiérarchisation des gestes de stabilisation. Chez les patients présentant une fracture de l'anneau pelvien, la fixation externe doit être faite en premier avant la fixation fémorale car il s'agit souvent de fractures instables et il est essentiel de stabiliser le patient en premier, la fixation externe fournit aussi la stabilité relative de l'anneau pelvien et facilite l'ostéosynthèse du fémur. Par contre chez les patients ayant une fracture du cotyle, les fractures fémorales doivent être fixées en premier pour pouvoir réduire ultérieurement plus facilement le cotyle. Nous rapportons le cas d'une patiente de 35 ans, victime d'une chute du 2^ème^ étage avec point d'impact au niveau de la hanche gauche. Le bilan lésionnel a objectivé des lésions traumatiques graves du bassin, comprenant une fracture du bassin type C3 selon la classification de Tile (lésions antérieures et postérieures avec instabilités rotatoire et verticale + fracture transversale du cotyle), une fracture du sacrum, de l'apophyse transverse de L5, du cadre obturateur controlatéral et une fracture ipsilatérale sous trochantérienne complexe réalisant le tableau de «hanche flottante». Nous avons préconisé d'abord la réduction et l'ostéosynthèse interne de l'extrémité supérieure du fémur (Clou Gama long) puis traitement des fractures du cotyle et du bassin.

**Figure 1:**
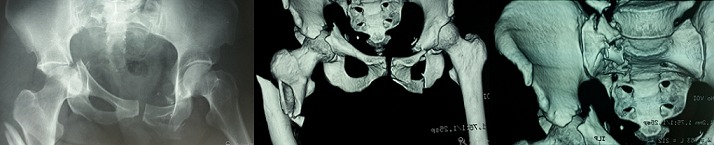
(A) radiographie du bassin face; (B) TDM du bassin avec reconstruction 3D; (C) TDM bassin montrant la fracture du cotyle, du sacrum et du rachis

